# Advanced Cardiac Imaging in the Assessment of Aortic Stenosis

**DOI:** 10.3390/jcdd10050216

**Published:** 2023-05-14

**Authors:** Kajetan Grodecki, Mateusz Warniello, Mateusz Spiewak, Jacek Kwiecinski

**Affiliations:** 1 1st Department of Cardiology, Medical University of Warsaw, Banacha 1a, 02-097 Warsaw, Poland; 2 Department of Interventional Cardiology and Angiology, Institute of Cardiology, Alpejska 42, 04-628 Warsaw, Poland; 3 Magnetic Resonance Unit, Department of Radiology, Institute of Cardiology, Alpejska 42, 04-628 Warsaw, Poland

**Keywords:** aortic stenosis, CT, CMR, PET, ^18^F-NaF

## Abstract

Aortic stenosis is the most common form of valve disease in the Western world and a major healthcare burden. Although echocardiography remains the central modality for the diagnosis and assessment of aortic stenosis, recently, advanced cardiac imaging with cardiovascular magnetic resonance, computed tomography, and positron emission tomography have provided invaluable pathological insights that may guide the personalized management of the disease. In this review, we discuss applications of these novel non-invasive imaging modalities for establishing the diagnosis, monitoring disease progression, and eventually planning the invasive treatment of aortic stenosis.

## 1. Introduction

Aortic stenosis (AS) is the most common form of valve disease in the Western world and a major healthcare burden that, with an ageing population, is projected to double by 2050. It affects up to 2% of patients over the age of 65. AS is characterized by progressive valve narrowing. The highly specialized leaflets that form the valve and must be compliant enough to open without resistance to blood flow become stiff and lose compliance. This results in an increase in the intraventricular pressure required to maintain the flow across the valve, which ultimately leads to myocardial decompensation and triggers patient symptoms [1,2]. Despite the high disease prevalence, there are no medical therapies to halt or delay disease progression, and the only available treatment is aortic valve replacement or implantation. The latter is typically performed with a bioprosthetic valve, which again is prone to degeneration and consequently requires careful surveillance to detect bioprosthesis failure in its early stages.

Given the burden of AS and the limited therapeutic options for treatment, there is a great unmet need for a better understanding of aortic valve pathophysiology. Such insights appear to be necessary to facilitate the development of new treatment strategies. The process of valve degeneration involves lipid deposition, inflammation, and calcification. The latter represents a self-perpetuating cycle of calcium formation (calcium salt precipitation) and valvular injury in which disease progression is dictated by the relentless accumulation of calcium in the valve leaflets [3,4]. Beyond valve degeneration, AS leads to a hypertrophic response in the myocardium that initially maintains cardiac performance but is eventually maladaptive, leading to decompensation, heart failure, and ultimately death [5,6]. Indeed, there is accumulating evidence that increasing levels of hypertrophy are maladaptive. Importantly in AS, patients display a marked variation in the magnitude of their hypertrophic response. This has prognostic implications and might explain the pronounced heterogeneity between symptom onset and the severity of valve narrowing in echocardiography [7].

Cardiac imaging plays a key role in the assessment of AS [8,9]. Echocardiography is the gold standard for evaluating the transvalvular flow and is used to grade the degree of stenosis (mild/moderate/severe). These assessments guide therapeutic decisions as patients with severe AS and symptoms of left ventricular decompensation have a class I indication for valve replacement. While this echocardiography-based algorithm forms the bedrock of clinical practice, recently, advanced cardiac imaging with cardiovascular magnetic resonance (CMR), computed tomography (CT), and positron emission tomography (PET) have provided invaluable pathological insights which have the potential to enhance the management of AS [8,10].

## 2. Cardiovascular Magnetic Resonance Imaging

State-of-the-art CMR can be leveraged for multiparametric heart evaluation. While CMR enables assessments of valvular pathology, it is particularly robust in depicting left ventricular remodelling, which ensues as a result of the increased afterload [11]. These assessments are recentered upon measuring the left ventricular (LV) mass, myocardial fibrosis, or systolic function. The most basic advantages of CMR are the lack of radiation exposure and more precise measurements compared to echocardiography. On the other hand, the cost and contrast administration should be considered as limitations [12]. While clinical AS work-up is focused on assessments of the severity of valvular narrowing, the role of adverse remodelling is garnering increasing interest. In particular, the presence and extent of myocardial fibrosis have been reinvigorated. The importance of that matter stems from the fact that once replacement myocardial fibrosis occurs, it is irreversible and acts as a predictor of death and unplanned cardiovascular hospitalizations in AS patients [13]. Because of this, the detection of replacement and diffuse fibrosis through CMR by using such tools as late gadolinium enhancement (LGE) and extracellular volume fraction (ECV) could potentially be used as a marker for early valve intervention [14].

Despite histological assessment of ventricular remodelling being the gold standard diagnostic tool, an invasive biopsy is limited to a small tissue sample, whereas CMR depicts the entire heart, facilitating a more global myocardium analysis [15]. Moreover, the multiparametric approach, including ECV and LGE, is optimal in comparison with biopsy not only because of safety but, more importantly, it allows better phenotyping of AS patients according to their myocardial response to AS in terms of myocardial fibrosis and morphological and functional cardiac alterations [16].

Adaptation of the left ventricle triggered by AS includes distinct patterns which may have an impact on prognosis. Dweck et al. described six patterns of ventricular remodelling and hypertrophy according to parameters measured in CMR, including LV mass, LV end-diastolic volume (LVDEDV), and mass/volume ratio M/V [17]. Beyond the normal ventricular structure, there are two forms of remodelling—concentric (with increased M/V with normal LV mass index) or asymmetric (with asymmetric wall thickening) and three models of hypertrophy: concentric (increased M/V, LV mass index), asymmetric (similar to concentric hypertrophy but with evidence of asymmetric wall thickening), and eccentric hypertrophy: (increased LV mass index, a dilated left ventricle, normal M/V, and a normal ejection fraction). A study showed no correlation between the severity of AS and certain morphological patterns of remodelling or hypertrophy, meaning that a normal LV structure can be observed amongst patients with severe stenosis, as LVH can coexist with moderate AS. These findings might explain the disconnection between the severity of AS and symptom onset. This is crucial since many studies suggest that the hypertrophic response within the myocardium is linked to mid-wall replacement fibrosis, which, as mentioned above, acts as an independent predictor of all-cause mortality in patients with moderate and severe disease [18]. These intriguing findings have been further explored in an outcome study involving 166 patients with moderate or severe AS. This analysis established that patients with asymmetric wall thickening had evidence of more advanced left ventricular decompensation with elevated myocardial injury and strain compared to those with concentric wall thickening. Additionally, subjects with asymmetric wall thickening had an adverse prognosis, with this form of remodelling acting as an independent predictor of aortic valve replacement or death after correction for age, sex, left ventricular mass index, coronary artery disease, and AS severity [19].

More advanced assessment, including left ventricular remodelling patterns in asymptomatic patients, may possibly halt the irreversible processes of fibrosis thanks to early intervention. Therefore, more research on CMR in AS is needed in order to develop a wider perspective on early AVR indications.

**Figure 1:**
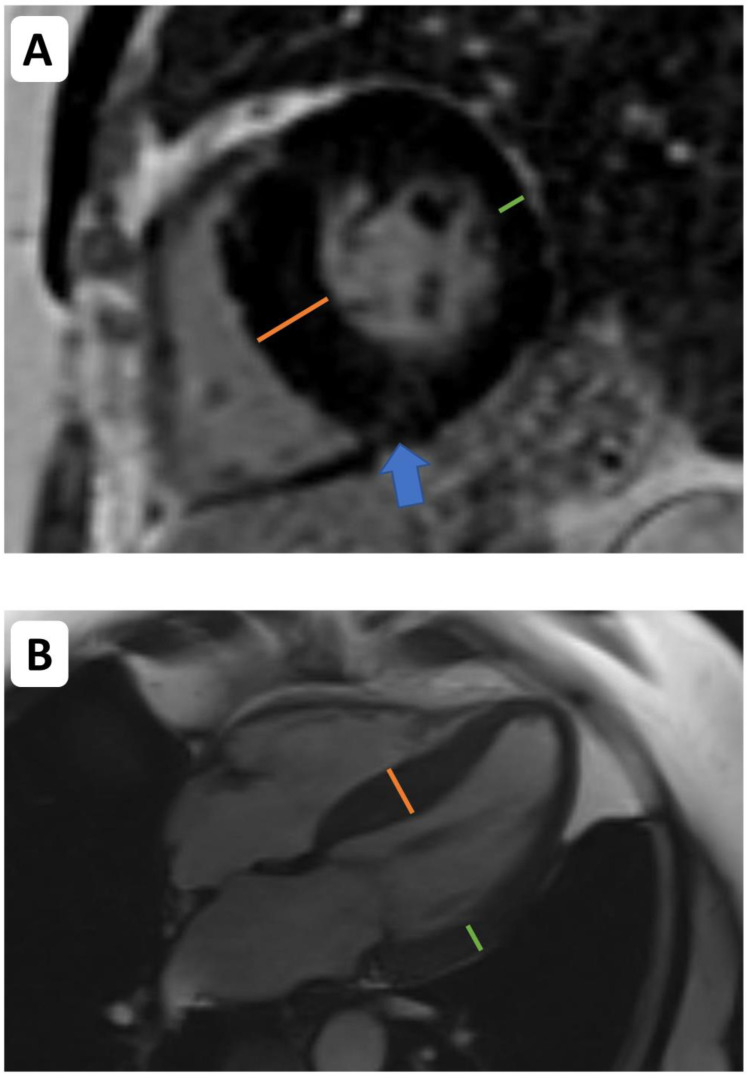
Cardiovascular magnetic resonance (CMR) imaging in the assessment of left ventricular remodelling in aortic stenosis. CMR facilitates assessments of left ventricular volumes, mass, wall thickness, and tissue characterization. Asymmetric wall thickening ((**A**,**B**)—the orange line indicates the abnormally thickened intraventricular septum. The green line indicates thickness of the opposing lateral wall). Midwall delayed enhancement (consistent with replacement fibrosis)—blue arrow indicates patchy midwall delayed enhancement within the insertion point of the right ventricle, which is often observed as a manifestation of left ventricular decompensation in pressure overload.

It has been established that CMR detects ventricular decompensation in AS through the identification of diffuse and replacement fibrosis (Figure 1). Chin et al. used T1 mapping to detect diffuse fibrosis associated with extracellular volume expansion and LGE to find replacement fibrosis [20]. By using LGE and T1 mapping techniques—ECV fraction and iECV (indexed extracellular volume)—it was possible to categorize patients into three different groups according to the level of myocardial fibrosis (normal myocardium, extracellular expansion, and replacement mid-wall fibrosis). This study concluded that there is evidence of increasing hypertrophy, myocardial injury, diastolic dysfunction, and longitudinal systolic dysfunction consistent with progressive left ventricular decompensation across the predefined groups. In this study, this categorization had prognostic implications, as a stepwise increase in unadjusted all-cause mortality was apparent between the aforementioned groups (8 deaths/1000 patient-years vs. 36 deaths/1000 patient-years vs. 71 deaths/1000 patient-years).

While echocardiography remains the gold standard for the assessment of transvalvular flows, state-of-the-art CMR imaging with phase-contrast velocity mapping can provide complementary findings. By using three-directional velocity encoding acquired over the entire cardiac cycle, CMR can facilitate measuring the flows in mixed aortic disease, such as a combination of aortic regurgitation and stenosis.

Cardiovascular magnetic resonance is not only a useful tool to quantify myocardial fibrosis but also to establish the differences between sex in the LV remodelling response to pressure overload [21]. In a multicenter study, T1 mapping was employed to measure the ECV fraction and quantify diffuse fibrosis, and LGE was leveraged to assess focal fibrosis; a group of male and female patients with moderate or severe AS were examined. The analysis showed that the female sex is related to higher ECV fraction and LGE despite having a better cardiovascular risk profile (with a lower prevalence of hypertension, dyslipidemia, diabetes or coronary artery disease). Thus, incorporating CMR into the AS diagnostic process might facilitate a more targeted therapeutic approach that could be offered depending on the sex of the patient [22].

With regard to left ventricle evaluation, CMR can deliver a great deal of information regarding the state of the myocardium, including the preoperative stage and its recovery after aortic valve replacement. Successful intervention plays a key role when it comes to clinical improvement in patients with severe AS [23,24]. Nevertheless, the extent of that improvement varies depending upon the exposure of the LV myocardium to the increased afterload before surgery [25,26]. Knowing that the extent of myocardial fibrosis as well as the overall degree of myocyte degeneration are inversely associated with both the systolic and diastolic left ventricular function, the presence of myocardial fibrosis might be a referral parameter in postprocedural patients’ reevaluation [15,25,26,27,28,29]. This is especially true since patients with irreversible LV dysfunction after surgery exhibit a substantially higher burden of replacement fibrosis [25]. For this reason, the prognostic significance of CMR in the increasing number of post-AVR patients should be widely acknowledged.

## 3. Computed Tomography

Computer tomography (CT) complements echocardiography in the diagnosis of AS and remains a major modality for the planning of transcatheter therapies. CT is characterized by superior spatial and temporal resolution, and therefore provides the best assessment of valvular calcifications among all imaging modalities.

Noncontrast CT is routinely used to quantify aortic valve calcium using the Agatston score, which takes into account both the density and the extent of calcium within the aortic valve complex. The attenuation range of 130 to 199 is assigned a weight of 1, while 200 to 299 is given a weight of 2, 300 to 399 is given a weight of 3, and any attenuation greater than 400 is given a weight of 4. To calculate the Agatston score, the lesion area is multiplied by an attenuation factor based on the highest Hounsfield units measured within that area [30]. This technique emerged as a robust tool supporting the diagnosis of severe AS in patients in whom echocardiography is inconclusive and has been endorsed by the European guidelines for the management of patients with valvular heart disease [31]. Optimal thresholding for severe stenosis was derived in a cohort of 646 patients with at least moderate AS and good left ventricular function who had undergone both echocardiography and CT calcium scoring [32]. Sex-specific thresholds of 1275 Arbitrary Units (AU) for women and 2065 AU in men were identified and validated in further studies proving that the aortic valve calcium score is a powerful biomarker predicting severe disease as well as one-year mortality in untreated patients [33,34].

**Figure 2:**
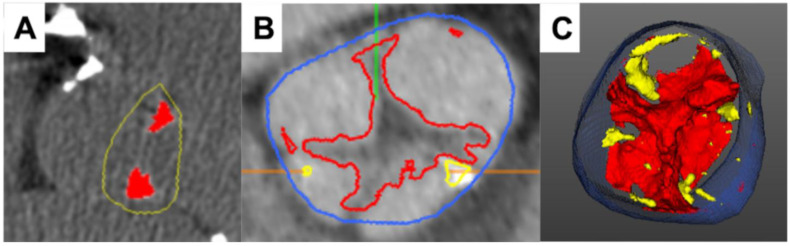
**Characterization of aortic valve composition via cardiac computed tomography.** Case example of a stenotic aortic valve with fibrotic tissue alterations, which are more pronounced than calcification ((**A**)—calcium score of 541AU). Computed tomography angiography ((**B**,**C**) allows for quantification of both fibrotic (red) and calcific (yellow) tissue components, improving the diagnostic accuracy of the modality (blue marks the aortic lumen; green and orange lines are software markers).

While AS in its end stage is predominantly associated with calcific alterations of valvular tissue, in a minority of patients, the fibrotic process may be more pronounced, resulting in leaflet thickening with a reduction in the aortic valve area and minimal accumulation of calcium [35]. Such presentation is especially common in younger patients with a bicuspid aortic valve. Therefore, the application of CT beyond a simple quantification of calcific tissue has been studied, and methods for the evaluation of full fibrocalcific composition from computed tomography angiography (CTA) have been recently proposed. Although differences in radiological density between respective tissue components are now often used for coronary plaque characterization, only pilot studies on its feasibility in AS exist so far [36,37,38,39]. The initial data suggested that the fibrocalcific volume of the aortic valve correlates with the ex vivo valve weight and is associated more closely with the peak aortic jet velocity than the Agatston score. Moreover, it has been demonstrated that quantifying the composition of aortic valve tissue from a CTA can improve the identification of severe AS and distinguish between high-gradient and low-flow low-gradient AS. While further studies are warranted, assessment of aortic valve tissue composition might increase the diagnostic accuracy of CTA (Figure 2).

Further, the three-dimensional nature of CT allows for precise evaluation of valvular anatomy, which may help overcome natural limitations of two-dimensional echocardiography, such as underestimation of the left ventricular outflow tract dimensions leading to discrepant severe AS grading [40,41]. A hybrid approach combining the true cross-sectional LVOT area from CT and Doppler hemodynamics in the continuity equation has been initially proposed by Kamperidis et al. [42]. It enabled the down-classifying of 52% of patients with normal flow low-gradient severe AS and 12% of patients with low-flow low-gradient severe AS and preserved LVEF as having moderate AS. The application of CTA might also improve the diagnostic certainty in discerning bicuspid aortic valves compared with echocardiography [43]. CT, especially when both the systolic and diastolic phases of the cardiac cycle are evaluated, offers diagnostic accuracy for valve types similar to intraoperative assessment [44]. For echocardiography, identification of bicuspid valves is limited by extensive calcifications causing acoustic shadows and suboptimal acoustic windows [45]. 

For these reasons, CT has emerged as a gold standard tool for planning transcatheter aortic valve implantation (TAVI), being endorsed by multiple societies [31,46]. Currently, CT is preferred over echocardiography for measuring the annulus to ensure accurate device sizing. Since the aortic annulus is a dynamic structure with dimensions being affected by the cardiac cycle, two-dimensional echocardiographic imaging may not provide enough precision required during pre-procedural work-up [47]. Indeed, the Pivotal Trial of Medtronic Corevalve and the PARTNER 3 trial have shown that using CT to size the annulus decreased the incidence of paravalvular leakages [48,49]. Moreover, highly reproducible tomographic measurements offer a more comprehensive understanding of the annular geometry, preventing potentially life-threatening complications such as coronary occlusions [50]. CT also facilitates the process of implantation by defining patients-specific ‘optimal’ C-arm angulations that directly reduce the time and radiation doses during the actual procedure [51]. Recognition of the device landing zone allows for qualitative grading of the calcifications that could potentially lead to annulus rupture, suboptimal expansions of the bioprosthesis, or post-procedural conduction disturbances and proceed with appropriate preemptive measures. The detailed measurements obtained by means of CTA can be leveraged for 3D printing of the valve apparatus—providing an opportunity for testing treatment options ex vivo. Beyond assessments of the aortic valve, CTA can evaluate the presence, severity, and extent of coronary artery disease in patients undergoing TAVI. Additionally, CTA has been demonstrated as a useful modality in a post-procedural follow-up, especially in patients with suspected subclinical leaflet thrombosis defined as hypoattenuating leaflet thickening with or without reduced leaflet motion [52]. This imaging phenomenon is observed in approximately 10% of bioprosthetic valves (both transcatheter and surgical) and was initially feared due to the risk of progression to clinical thrombosis or potential association with cerebral indicants; current data suggest a rather benign nature of this phenomenon [53,54]. Nevertheless, the long-term impact of leaflet thickening on valve durability is yet to be determined. 

Finally, CTA could be a useful tool in the assessment of left ventricle geometry and function. Undoubtedly, echocardiography is essential in assessing the function of AS patients both before and after medical interventions. Nonetheless, image quality can be impaired in this elderly population due to restricted planes, acoustic shadowing, and patient-specific factors. Evaluating left ventricular global longitudinal strain using dynamic feature tracking CT data has been shown to be achievable in patients with AS [55]. However, compared to speckle-tracking echocardiography, feature tracking with CT tends to underestimate global longitudinal strain values [56]. The source of this discrepancy could be potentially attributed to differences in temporal resolutions of CT and echocardiography as well as mathematical principles for the calculation of strain values in both modalities. Considering the important prognostic role of left ventricular function parameters, their derivation from routinely performed CT scans may greatly improve the importance of this imaging modality in the personalization and timing of treatment in AS [57].

## 4. Positron Emission Tomography

While CT and transthoracic echocardiography can identify the structural and haemodynamic manifestations of valvular disease, they do not offer insights regarding the molecular processes driving valve disease [58]. These can be characterized using Raman spectroscopy—a promising, highly sensitive technique used to assess the topographical distribution of chemical elements to complex biomolecules in cultured cells, tissues, and organs that has proven to provide reliable fingerprints of various diseases in vivo, as well as those concerning the cardiovascular system [59,60,61]. Of note, Raman spectroscopy revealed calcium hydroxyapatite colocalized with clustered lipid derivatives and carotenoids in ex vivo aortic valve leaflets affected by calcific AS [62].

Given that calcification plays a central role in aortic valve degeneration beyond imaging calcium, there is increasing interest in imaging developing calcifications. Measuring the activity of calcification processes can be achieved with ^18^F-sodium fluoride (^18^F-NaF) PET [63]. This technology was traditionally used for imaging bone malignancies as it depicts areas of rapid bone turnover. In whole-body PET, it was soon discovered that tracer uptake could be found within the cardiovascular structures [64]. Initial studies showed an intriguing association between atherosclerotic plaque morphology and ^18^F-NaF activity [65]. While established calcified lesions demonstrated no uptake, partially calcified and noncalcified plaque were more often ^18^F-NaF avid [66,67]. Further studies showed that ^18^F-NaF uptake is a hallmark of lesions with unfavourable morphology, those that are rapidly progressing, and ruptured culprit plaques in patients with recent myocardial infarction [68,69,70,71,72,73]. In parallel to studies focused on ^18^F-NaF atherosclerotic plaque imaging, researchers have evaluated the utility of non-invasive active calcification processes’ imaging in the context of valvular disease [74].

Dweck et al. showed that among patients with AS, 91% had increased ^18^F-NaF uptake, with a stepwise increase in tracer activity across the spectrum of AS severity [75]. Furthermore, a longitudinal study demonstrated that baseline ^18^F-NaF uptake is a predictor of AS progression. On follow-up CT scans performed 1 year or 2 years following baseline PET, aortic valve calcification increased [76,77]. Baseline ^18^F-NaF uptake is closely associated with the calcium score change and was primarily observed within previously noncalcified regions. New valvular calcium on CT was detected at similar locations as the ^18^F-NaF activity on baseline PET imaging. Moreover, on a patient level, calcium buildup progressed three times more rapidly in participants with high ^18^F-NaF uptake.

The ability to monitor disease activity within the aortic valve non-invasively with PET has been leveraged in clinical trials that aim to test novel medication for halting AS progression. Such an approach has been employed in the context of the Study Investigating the Effect of Drugs Used to Treat Osteoporosis on the Progression of Calcific Aortic Stenosis (SALTIRE II) [78]. In a double-blind, randomized controlled trial, Pawade et al. tested whether drugs targeting processes of active calcification—denosumab or alendronic acid—could slow the progression of AS [78]. Although in 100 patients who were randomized to the intervention arm, effective inhibition of bone resorption was confirmed (on the basis of the release of carboxy-terminal telopeptides of fibrillar collagens into the circulation), there were no changes in the magnitude of progression of aortic valve calcification or function over the 2 years of follow-up between the experimental and placebo arms [78]. Neither denosumab nor alendronate led to major amelioration or facilitated an acceleration of aortic valve calcification or disease progression, as evidenced by 18F-sodium fluoride positron emission tomography, computed tomography, or echocardiography. Imaging microcalcification with ^18^F-NaF has also been leveraged in the Bicuspid Aortic Valve Stenosis and the Effect of vitamin K2 on Calcium metabolism on ^18^F-NaF PET/MRI (BASIK2) trial and in the Progression of Aortic Stenosis trials, which tests the utility of PCSK9 Inhibitors [79,80]. Both these trials are ongoing and will soon report their findings.

**Figure 3:**
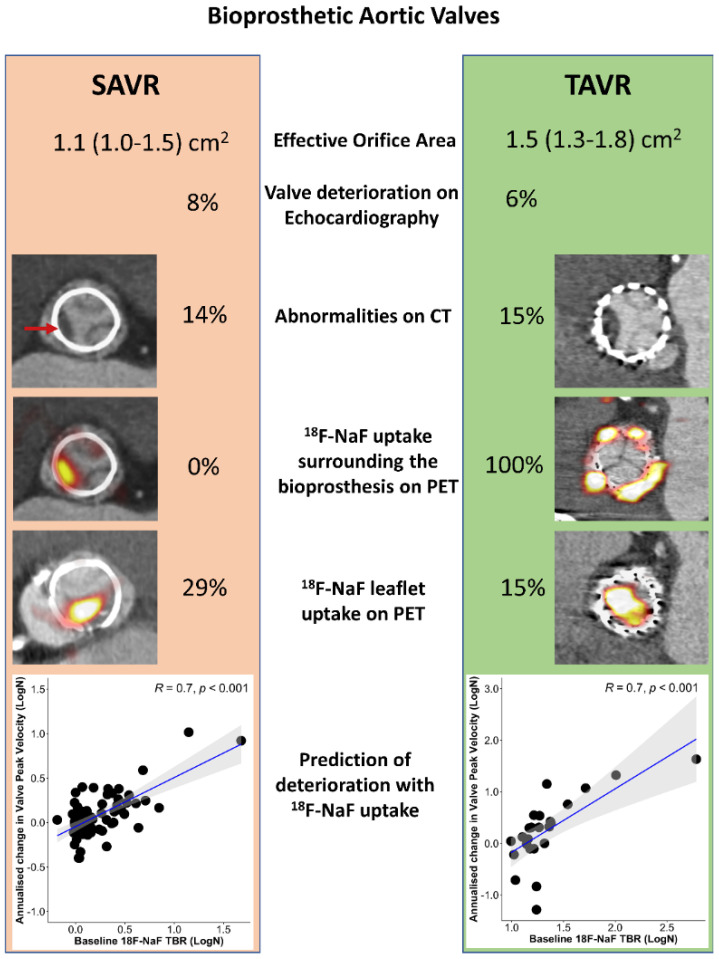
Imaging aortic bioprosthesis valve failure. Echocardiographic, computed tomography (CT), and ^18^F-sodium fluoride (^18^F-NaF) findings in 47 patients with transcatheter aortic valve replacement (TAVR) with 51 patients with surgical aortic valve replacement (SAVR) who underwent the same research imaging protocol. ^18^F-NaF uptake was observed on the peripheral of all TAVR valves and none of the SAVR valves. While patients with TAVR showed lower peak velocity (2.4 (2.0–2.7) [61] vs. 2.7 (2.4–3.0) m/s, *p* = 0.03) and larger effective orifice area (1.5 (1.3–1.8) vs. 1.1 (1.0–1.5) cm^2^, *p* = 0.02) than patients with SAVR, baseline echocardiographic (6 vs. 8% *p* = 0.78) and CT abnormalities (15 vs. 14% *p* = 0.87) suggestive of bioprosthetic degeneration were detected in a similar proportion of patients with either TAVR or SAVR (red arrow). The overall prevalence of patients with increased leaflet ^18^F-NaF uptake was nearly double in patients with SAVR compared to those with TAVR (29% and 15%, *p* = 0.09). In both patients with SAVR or TAVR, baseline ^18^F-NaF leaflet uptake was predictive of the change in the peak transvalvular velocity on echocardiography (scatterplots display the annualized change in the peak velocity with the line of the best fit in blue and the confidence intervals in grey). Reprinted from *CIRCULATION*, Vol. 144, Issue 17, Kwiecinski et al., Native Aortic Valve Disease Progression and Bioprosthetic Valve Degeneration in Patients With Transcatheter Aortic Valve Implantation, pages 1396–1408, 2021 [82], with permissions from Wolters Kluwer.

Beyond imaging native aortic valve disease, ^18^F-NaF PET lends itself to imaging patients following valve replacement (Figure 3). As bioprosthetic aortic valves are prone to degeneration, tools for detecting early stages of dysfunction or morphological abnormalities are needed. The current standard of care relies on clinical assessment and serial echocardiography, which are aimed at detecting the valve dysfunction that occurs only toward the end stages of the degeneration process [81]. As a result, the patient typically presents with overt bioprosthetic valve failure, which is often a life-threatening state [81]. Given that calcification plays an important role in the process of bioprosthesis degeneration, ^18^F-NaF PET has been evaluated as a tool for identifying patients in the early stages of the disease. In a series of observational studies, it was demonstrated that ^18^F-NaF uptake acts as an early independent indicator of future bioprosthetic valve failure [81,82]. Importantly, ^18^F-NaF PET outperformed all other predictors, including the valve type and age, and echocardiographic and CT findings in detecting early bioprosthetic valve degeneration and predicting future valve dysfunction uptake. The authors therefore concluded that ^18^F-NaF PET provides a readily applicable tool for measuring disease activity within the valve, which has the potential to transform how we monitor and treat the expanding population of patients living with bioprosthetic valves [81,82].

The encouraging results of the studies discussed above have been facilitated by the advances in PET scanners and novel image acquisition, reconstruction, processing, and analysis protocols [83,84,85,86,87,88,89]. While these have been primarily developed for imaging coronary atherosclerosis, due to the similar size of the target and patterns of motion involving the aortic valve, they have been successfully employed for valvular ^18^F-NaF imaging. Correcting for cardiac motion, the delay of tracer injection to emission scanning has been shown to enhance the reproducibility of uptake assessments [90]. Similarly, optimized reconstruction protocols involving time of flight data and point spread function correction improve image quality [91,92]. Given the abundance of dedicated methods using the full potential of PET for imaging cardiovascular disease in the near future, we will witness further advances in molecular aortic valve imaging with PET [93,94,95,96,97].

## 5. Conclusions

In summary, advanced cardiac imaging has already provided invaluable insights into the pathophysiology of AS. Given the growing number of studies and trials that are currently ongoing, in the near future, further applications of CMR, CT, and PET in the context of AS will likely emerge. Ultimately, advanced cardiac imaging holds real clinical potential for refining how we manage patients with AS.

## Data Availability

Not applicable.
